# The Impact of Artificial Marble Wastes on Heat Deflection Temperature, Crystallization, and Impact Properties of Polybutylene Terephthalate

**DOI:** 10.3390/polym13234242

**Published:** 2021-12-03

**Authors:** Tianliang Feng, Yangzhou Li, Liang Fang, Zhenming Chen

**Affiliations:** 1 Guangxi Key Laboratory of Optical and Electronic Materials and Devices, College of Material Science and Engineering, Guilin University of Technology, Guilin 541004, China; fengtianliang001@aliyun.com (T.F.); liyangzhou0@aliyun.com (Y.L.); fanglianggl001@aliyun.com (L.F.); 2 Guangxi Key Laboratory of Calcium Carbonate Resources Comprehensive Utilization, College of Materials and Chemical Engineering, Hezhou University, Hezhou 542899, China

**Keywords:** polybutylene terephthalate, artificial marble wastes, melt blending, partial core-shell structure

## Abstract

As artificial marble is abundant and widely used in residential and commercial fields, the resource utilization of artificial marble wastes (AMWs) has become extremely important in order to protect the environment. In this paper, polybutylene terephthalate/artificial marble wastes (PBT/AMWs) composites were prepared by melt blending to maximize resource utilization and increase PBT performance. The research results showed that the filling of AMWs was beneficial to the improvement of PBT-related performance. X-ray diffraction analysis results indicated that after filling AMWs into the PBT matrix, the crystal structure of PBT was not changed. Heat deflection temperature (HDT) analysis results indicated that the HDT of PBT composites with 20 wt% AMWs reached 66.68 °C, which was 9.12 °C higher than that of neat PBT. Differential scanning calorimetry analysis results showed that heterogeneous nucleation could be well achieved when the filling content was 15 wt%; impact and scanning electron microscope analysis results showed that due to the partial core-shell structure of the AMWs, the impact strength of PBT was significantly improved after filling. When the filling amount was 20 wt%, the impact strength of the PBT composites reached 23.20 kJ/m^2^, which was 17.94 kJ/m^2^ higher than that of neat PBT. This research will not only provide new insights into the efficient and high-value utilization of AMWs, but also provide a good reference for improved applications of other polymers.

## 1. Introduction

Artificial marble is a non-degradable crosslinked polymer composite composed of calcium carbonate powder and unsaturated polyester [1]. Compared with natural marble, artificial marble not only has excellent processability, impact resistance, and corrosion resistance, but it also has unique aesthetic and technical characteristics, and is widely used for decoration in the residential and commercial fields [2,3,4]. Artificial marble wastes (AMWs) are a by-product of the production of artificial marble. As a result of its surface containing residual unsaturated polyesters, it is difficult for it to degrade in natural environments or undergo secondary processing [5,6]. If AMWs are not handled well, it is not only a waste of resources, but it also causes environmental pollution and poses a threat to people’s health [7,8,9]. Generally, most AMWs were disposed of in landfills, chemical cycles, and by combustion [10,11]. These methods usually have serious limitations, including inefficient utilization and causing new pollution problems [12]. Therefore, if AMWs are regarded as a kind of dispersion filler, it can produce interface effects with some polymer matrix to a certain extent. Buketov and stukhlyak et al. [13,14] found that these interface effects include the chemical interaction between filler and polymer matrix macromolecules, as well as directly between active side groups or segments of macromolecular chains, which can effectively enhance the mechanical properties of the polymer. Especially for impact strength, the addition of dispersion filler can improve the crack extension mode of the impact fracture, resulting in brittle–ductile fracture transition. At present, some work on the resource utilization of AMWs has been carried out. Huang et al. [15] filled AMWs into a polyvinyl alcohol (PVA) matrix as functional filler and added a prepared formamide/water composite plasticizer to improve the melt processability of PVA. Their results indicated that the composite plasticizer could promote the complexation between AMWs and PVA molecules. Due to the nucleation of AMWs, the grain size of PVA was significantly reduced, and the melting processing window was expanded. When 10% AMWs was added, the tensile strength of PVA/AMWs composites increased by 35.4%. Chen et al. [16] added AMWs into high-density polyethylene wood composites. The results showed that when the content of AMWs was 60%, the flexural strength and flexural modulus of the composites were increased by 12.9% and 80%, respectively. At the same time, when maleic anhydride grafted polyethylene was added as an adhesive, the notch impact strength and tensile strength of the composites reached 5.03 kJ/m^2^ and 28.0 MPa, respectively. By electron beam irradiation, Kim et al. [17] used carboxymethyl cellulose (CMC) and AMWs as precursor and filler, respectively, and prepared CMC/AMWs carbon foam by carbonization. The results indicated that the addition of AMWs increased the thermal stability of CMC and the carbon residue at 900 °C, and the CMC/AMWs carbon foam also showed good compressive strength and thermal conductivity. Therefore, it is necessary to reuse and recycle AMWs in an effective and environmentally friendly manner.

Polybutylene terephthalate (PBT) is well known as one of the five most commonly used plastics due to its remarkable comprehensive properties, such as its excellent mechanical properties, corrosion resistance, and electrical properties. As a result, it is widely used in electronics and electrical appliances, the automobile industry and its related machinery, instruments, and household appliances [18,19,20,21,22]. However, its disadvantages of low heat deflection temperature and high impact sensitivity limit its development for some applications and require significant improvement [23,24,25,26,27]. In general, calcium carbonate [28], glass fibers [29,30,31,32], carbon fibers [33], montmorillonite [34], and carbon nanotubes [35,36] are commonly used as PBT reinforcements [37].

In the present research, we describe the preparation process of AMWs-filled PBT composites, and investigate their comprehensive properties. The main purpose of this work was to test the effects of AMWs on the heat deflection temperature, crystallization, and impact strength of PBT/AMWs composites, in order to provide a good reference for the resource utilization of AMWs and the preparation of high-performance polymer matrix composites.

## 2. Materials and Methods

### 2.1. Materials

AMWs, which were collected from the cutting process of artificial marble production, were provided by Guangxi Lisheng Stone Industry Co., Ltd., (Hezhou, China). PBT resin with a trade name of S600F20 was purchased from Wanhua Chemical Group Corp., Ltd., (Yantai, China).

### 2.2. Sample Preparation

#### 2.2.1. Pretreatment of AMWs

**Figure 1:**
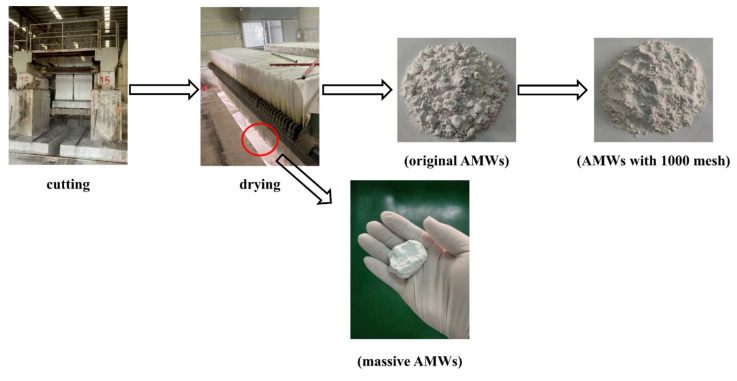
Schematic illustration of pretreatment of AMWs.

In order to reduce the uncertainty of large AMWs particles on the experimental results, a 1000-mesh wet sieving treatment was carried out for the AMWs prior to the experiments (Figure 1).

#### 2.2.2. Preparation of PBT/AMWs Composites

Compared with our previous work, the composites prepared by in-situ polymerization of PBT and AMWs with low filling content had a less obvious improvement in comprehensive properties and did not achieve the originally-expected effect. The melt blending had a better mixing effect and could promote compatibility between components, it could achieve the preparation of PBT/AMWs composites with high AMWs filling content, which may have further enhanced the effect of AMWs on the comprehensive properties of PBT. The AMWs particles were first dried at 120 °C for 10 h. Then, PBT and AMWs were mixed in an internal mixer (ZG-0.2LJ; Dongguan Zhenggong Electromechanical Equipment Co., Ltd., Dongguan, China). After that, the mixture was extruded in a micro-conical, twin-screw extruder (SJZS-10B; Wuhan Ruiming Experimental Instrument Co., Ltd., Wuhan, China) at a temperature between 260 and 280 °C. After granulation, the extruded blend was dried for 8 h and injection molded into three-dimensional samples (80 mm × 10 mm × 4 mm) using a micro injection machine at a melting temperature of 280 °C (SZS-20; Wuhan Ruiming Experimental Instrument Co., Ltd., Wuhan, China).

The formulations used to prepare the PBT/AMWs composites are shown in Table 1. The naming rule was PBT-X, where X represented the filling proportion (weight percentage) of the AMWs.

### 2.3. Characterization of PBT and PBT/AMWs Composites

#### 2.3.1. Particle Size Distribution

The particle size distribution of the AMWs before and after sieving was determined by a BT-2600 laser particle size distribution instrument (Dandong Baite instrument Co., Ltd., Dandong, China). The sampling time was 800, and the cycle speed was 1600 rpm.

#### 2.3.2. Scanning Electron Microscopy (SEM)

Scanning electron microscopy (SEM) observation was performed on the surface morphology of the AMWs before and after sieving and fracture surface of impact samples. The Surfaces were sprayed with gold and observed using a JSM-7610F field emission scanning electron microscope (JEOL Corp., Tokyo, Japan) under a 20 kV accelerating voltage.

#### 2.3.3. Thermogravimetric Analysis (TGA)

Thermogravimetric analysis (TGA) measurements were performed on a TGA4000 thermal analyzer (PE Corp., Waltham, MA, USA). The temperature ranged from 30 to 900 °C at 10 °C/min in a nitrogen atmosphere at an exchange rate of 20 mL/min.

#### 2.3.4. X-ray Diffraction (XRD)

X-ray Diffraction (XRD) patterns were measured with a Rigaku Ultima IV diffractometer (Rigaku Corp., Tokyo, Japan). A CuK_α_ wavelength of 0.154 nm was used, and the scanning range was 5–80° with a 10°/min scanning rate.

#### 2.3.5. Heat Deflection Temperature (HDT)

The heat deflection temperature (HDT) values of the samples (80 mm × 10 mm × 4 mm) were obtained by an HDT/V-1103 heat distortion and vicat softening temperature instrument (Chengde Jinjian Testing Instrument Co., Ltd., Chengde, China). The samples were placed under a specific load of 1.8 MPa according to the ISO 75 standard, and all the HDT values were the average values of five tests.

#### 2.3.6. Differential Scanning Calorimetry (DSC)

Differential scanning calorimetry (DSC) measurements were performed with a DSC25 instruments device (TA Corp., New Castle, DE, USA). The melting and crystallization behaviors of all the samples were investigated by the following procedure in a nitrogen atmosphere at an exchange rate of 50 mL/min: the dried samples were first heated at a heating rate of 20 °C/min from 0 to 280 °C, and then held for 2 min before cooling to 30 °C at a rate of 20 °C/min; at the end of each cooling, the samples were held for 2 min and then heated to 280 °C at a rate of 10 °C/min. The cooling and heating processes were recorded.

#### 2.3.7. Impact Strength

The impact strength values of the samples (80 mm × 10 mm × 4 mm) were recorded by a CEAST 9050 Pendulum impact testing machine (Boston, MA, USA). The energy of the pendulum was 5.5 J according to the ISO 180 standard, and all the impact strength values were the average values of five tests.

## 3. Results and Discussion

### 3.1. Characterization of AMWs

**Figure 2:**
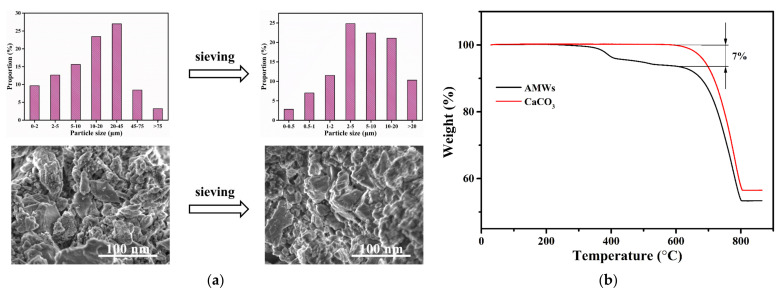
(**a**) Particle size distribution and SEM images before and after sieving of AMWs; (**b**) TGA curves of AMWs and CaCO_3_.

The AMWs were wet-sieved with a 1000-mesh in the pretreatment, and their particle size distribution and SEM images are shown in Figure 2a. From the particle size distribution before and after AMWs sieving in Figure 2a, it was clear that the original AMWs particle size was larger and the distribution interval was relatively dispersed, while the particle size decreased significantly and the distribution interval became concentrated after sieving. According to the particle size test results, the average diameters of the AMWs before and after sieving were 20.95 and 8.875 μm, respectively. As can be seen from the SEM images before and after AMWs sieving in Figure 2a, the shape of the AMWs particles became relatively uniform. TGA curves of the AMWs and CaCO_3_ particles are shown in Figure 2b; approximately 7% of the weight loss in the range of 250~600 °C on the AMWs TGA curve was due to the thermal degradation of unsaturated polyester coated on its surface, which was consistent with the industry notion that artificial marble is mainly composed of approximately 93% heavy calcium powders/sands and 7% unsaturated polyesters.

### 3.2. XRD Analysis

**Figure 3:**
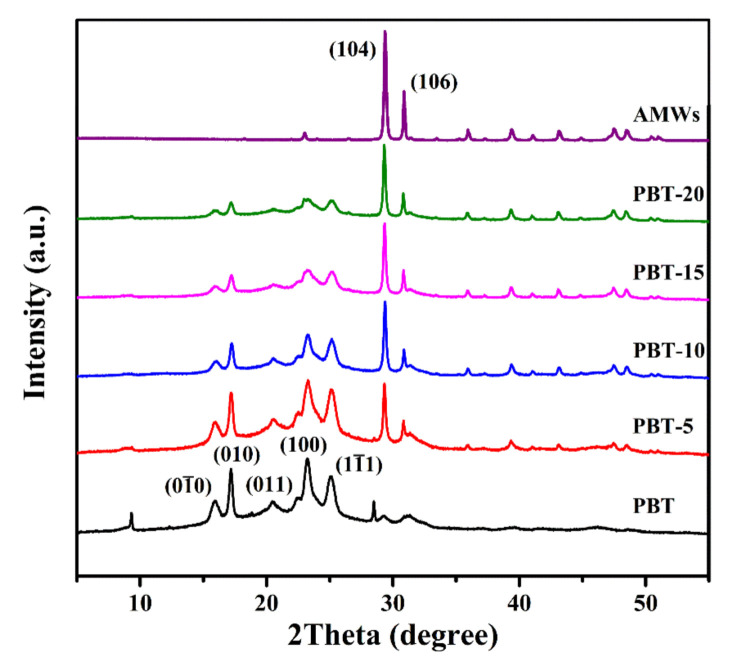
XRD patterns of PBT, PBT/AMWs composites and AMWs.

Figure 3 presents the XRD results of the PBT/AMWs composites. Five characteristic peaks were observed near the 2θ values of 15.8°, 17.1°, 20.4°, 23.1°, and 25.0°, which correspond to the reflections from the (010), (010), (011), (100) and (111) planes, respectively; this was indicative of the α-form of neat PBT [38]. At the same time, the intensity of the characteristic peaks of PBT gradually weakened as the filling amount of the AMWs increased. This could be attributed to that although filled AMWs played a role of heterogeneous nucleation, the dispersion of AMWs in the PBT matrix hindered the movement of PBT molecular chains, which affected the arrangement of PBT molecular chains, thereby reducing the crystallinity. Furthermore, characteristic 2θ peaks at 29.4° and 31.1° were assigned to the (104) and (006) crystal planes of the AMWs, respectively [15]. Therefore, the addition of AMWs had no effect on the crystal structure of PBT.

### 3.3. HDT Analysis

**Figure 4:**
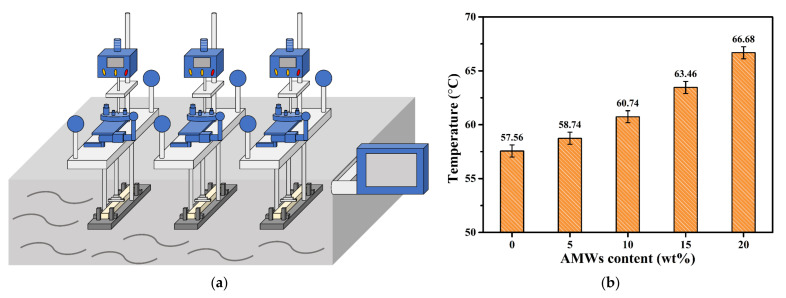
(**a**) Schematic illustration of the device used for HDT testing; (**b**) HDT of PBT and PBT/AMWs composites.

The device used for HDT testing is shown in Figure 4a. As is known, the definition of the HDT is the upper-limit temperature of dimensional stability without significant deformation under normal load and thermal effects during the use of a material; it is usually used as the highest continuous-use temperature in the material selection process in industry and can provide important information for material design [39,40,41]. It is evident from Figure 4b that in the absence of AMWs, the HDT of neat PBT was only 57.56 °C. As the filling amount of AMWs increased, the HDT of the PBT/AMWs composites gradually increased. When the filling amount was 20 wt%, the HDT reached 66.68 °C, which was 9.12 °C higher than that of neat PBT. This could be attributed to the filling amount of AMWs increasing the force between the PBT molecular chains, and inhibiting their movement, which resulted in an increased resistance to bending deformation, thereby hardening the matrix [42]. This has important practical significance for expanding the engineering applications of PBT.

### 3.4. DSC Analysis

**Figure 5:**
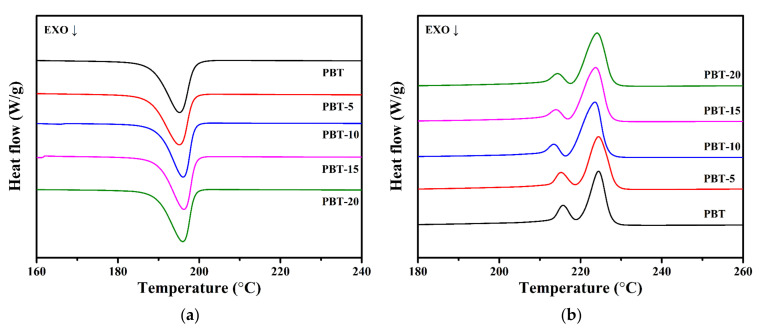
(**a**) DSC cooling and (**b**) second heating curves for PBT and PBT/AMWs composites.

Figure 5 illustrates the DSC curves of the cooling and second heating (after removing the thermal history due to processing–melt mixing conditions), and Table 2 summarizes the thermal parameters from the DSC curves.

Figure 5a shows that when the filling amount of AMWs increased, the crystallization peak temperature of the PBT/AMWs composites gradually shifted towards higher temperatures, and the crystallization peak width gradually decreased. When the filling amount exceeded 15 wt%, the crystallization peak temperature (*T*_c_) increased. This was because the AMWs had a high specific surface area after the sieving treatment, which significantly increased the nucleation point of the PBT molecular chains; 15 wt% AMWs had the best heterogeneous nucleation effect on the PBT crystals [43]. From Figure 5b, the melting peak temperature (*T*_m_) of the PBT/AMWs composites showed little change compared with neat PBT, indicating that the filling of AMWs had basically no effect on the crystal integrity of PBT or the thickness of the wafer.

### 3.5. Impact Strength Analysis

**Figure 6:**
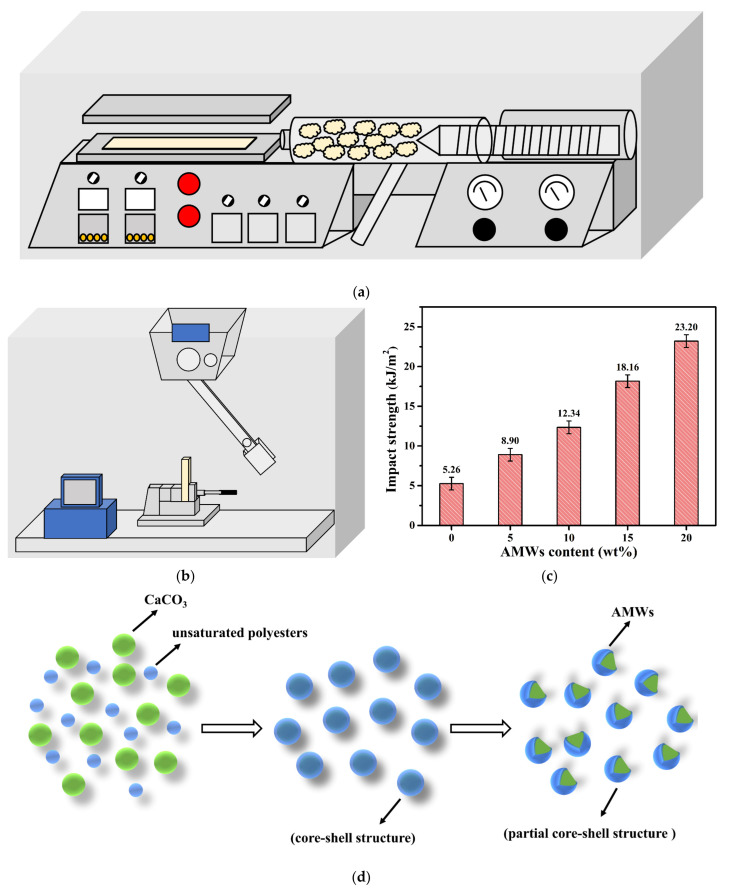
(**a**) Schematic illustration of injection; (**b**) schematic illustration of impact testing; (**c**) impact strength of PBT and PBT/AMWs composites; (**d**) schematic illustration of partial core-shell structure formation of AMWs.

Impact strength characterizes the ability of a material to absorb energy in the process of impact fracture, and is one of the indicators used to measure the toughness of a material [44]. As shown in Figure 6a, the three-dimensional impact samples were injection molded by a micro injection machine. Impact strength testing was carried out by the impact-testing machine in Figure 6b, and the corresponding results are shown in Figure 6c. The impact strength of neat PBT was only 5.26 kJ/m^2^, and the impact strength of the composite materials increased significantly with the increase in AMWs filling. When the AMWs filling was 20%, the impact strength reached 23.20 kJ/m^2^, which was 17.94 kJ/m^2^ higher than that of neat PBT. This could be attributed to the fact that the surface of the AMWs was coated partly by unsaturated polyesters, forming a special “partial core-shell structure”. The heavy calcium powder used in the production of artificial marble is usually 325 mesh at finest, and the AMWs used in this experiment passed 1000-mesh wet sieving, indicating that in the cutting process of artificial marble production, the initial heavy calcium powder was cut open again, and the new section produced had no coating (Figure 6d). Compared with the inner core, the unsaturated polyesters of the outer layer effectively protected the core when impacted by external forces. Therefore, this partial core-shell structure of the AMWs had a good toughening effect on the PBT matrix [45,46,47,48]. In addition, Jarzabek [49] and Alfano et al. [50] found that the interface effects between the added filler and the matrix were crucial for obtaining ideal composite materials with better mechanical properties. Since the unsaturated polyesters coated on the surface of the AMWs and the PBT matrix carried some same groups (the carbonyl groups, phenyl groups, and methylene groups), there was a structural similarity between the AMWs and the PBT matrix, which induced a high degree of molecular chain entanglement and strengthened the compatibility, leading to an increased viscosity of the entire system. Therefore, as the filling content of AMWs increased, the interface effects between the AMWs and the PBT matrix were continuously strengthened.

### 3.6. SEM Analysis

**Figure 7:**
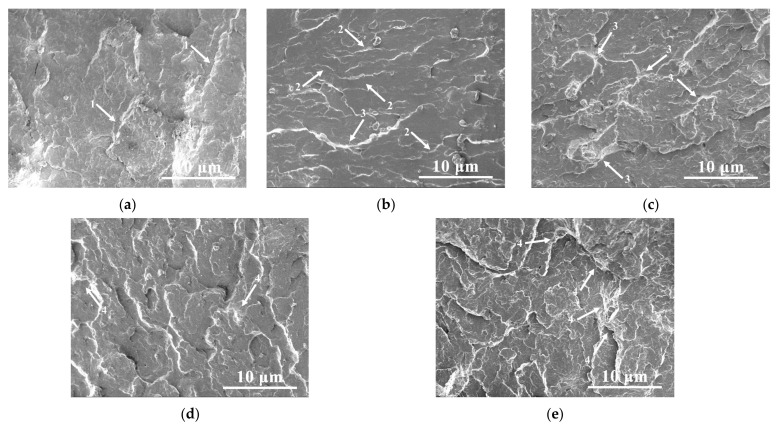
SEM images of the fractured surface of (**a**) PBT, (**b**) PBT-5, (**c**) PBT-10, (**d**) PBT-15, and (**e**) PBT-20.

Figure 7 shows SEM images of the impact sample fracture surface of neat PBT and the PBT/AMWs composites, and the viscus and brittle zones related to crack growth were marked with arrows. It can be seen from Figure 7a that the fracture surface of neat PBT was relatively flat and smooth, accompanied by the appearance of several single straight cracks (arrow 1), showing obvious brittle–fracture characteristics. When filled with 5% and 10% AMWs, microcracks (arrow 2) were gradually seen on the fracture surface (Figure 7b,c) accompanied by a few obvious ridges (arrow 3), and these viscus zones showed certain ductile–fracture characteristics. Upon further filling with AMWs, it was found that the fracture surface became rougher and crack growth revealed noticeable irregularity (Figure 7d,e), accompanied by the appearance of intricate dimple contours (arrow 4). In addition, the coalescence of adjacent ductile microcracks confirmed that the material ductility was increased, resulting in the increase in energy expended in fracture [51]. The ductile–fracture characteristics of the PBT-20 composite fracture surface were the most obvious; thus, it had the optimal impact performance. This also showed that when the PBT samples were impacted by an external force, AMWs could act as a stress concentrator and play a role in bearing the external force and absorbing energy.

## 4. Conclusions

Our research showed that AMWs could be used as fillers to fill PBT matrices and improve their related properties. XRD analysis results showed that filling AMWs into a PBT matrix would not change the crystal structure of PBT. HDT analysis results showed that when filled with 20 wt% AMWs, the HDT of PBT composites reached 66.68 °C, which was 9.12 °C higher than that of neat PBT. DSC analysis showed that heterogeneous nucleation could be well achieved when the filling content was 15 wt%; impact and SEM analysis results showed that due to the partial core-shell structure characteristics of the AMWs, the impact properties of PBT could be significantly improved after filling. When the filling amount was 20 wt%, the impact strength of the PBT composites reached 23.20 kJ/m^2^, which was 17.94 kJ/m^2^ higher than that of neat PBT. These findings revealed the great potential of filling AMWs to upgrade PBT in engineering applications. We believe that the strategy of using AMWs as filler to prepare composites will promote the efficient and high-value utilization of AMWs and provide a good reference for improved applications of other polymers.

## Figures and Tables

**Table 1:** The formulations of PBT and PBT/AMWs composites.

Sample	PBT (g)	AMWs (g)	AMWs (wt%)
PBT	400	0	0
PBT-5	380	20	5
PBT-10	360	40	10
PBT-15	340	60	15
PBT-20	320	80	20

**Table 2:** The thermal parameters from the DSC curves.

Samples	*T*_0_ (°C)	*T*_c_ (°C)	*T*_m_ (°C)
PBT	204.72	195.13	224.43
PBT-5	202.51	195.15	224.43
PBT-10	202.34	196.04	223.55
PBT-15	201.94	196.27	223.73
PBT-20	202.16	195.99	224.06

## Data Availability

The data presented in this study are available upon request from the corresponding author.
